# Bicyclist Deaths Associated with Motor Vehicle Traffic — United States, 1975–2012

**DOI:** 10.15585/mmwr.mm6431a1

**Published:** 2015-08-14

**Authors:** Jason Vargo, Benjamin G. Gerhardstein, Geoffrey P. Whitfield, Arthur Wendel

**Affiliations:** 1 Global Health Institute, University of Wisconsin-Madison; 2 Division of Community Health Investigations, Agency for Toxic Substances Disease Registry; 3 Healthy Community Design Initiative, National Center for Environmental Health, CDC

Physical activity, including bicycling, is linked with multiple health benefits (1). However, although bicycles account for only about 1% of trips across all modes of transportation, on a per trip basis, bicyclists die on U.S. roads at a rate double that of vehicle occupants (2). In 2009, an estimated 392 billion trips (across all modes) were taken in the United States, including 4.1 billion bicycle trips, and 33,808 deaths occurred on U.S roadways (across all modes), including 630 bicyclist deaths (3–5). This report examines mortality trends among cyclists using national collision data from the Fatality Analysis Reporting System (FARS) for the period 1975–2012. Annual rates for cyclist mortality decreased 44%, from 0.41 to 0.23 deaths per 100,000 during this period, with the steepest decline among children aged <15 years. In recent years, reductions in cyclist deaths have slowed. However, age-specific cyclist mortality rates for adults aged 35–74 years have increased since 1975. Multifaceted approaches to bicyclist safety have been shown to be effective in increasing bicycling while decreasing traffic injuries and fatalities (1). With U.S. adults choosing to walk and cycle more, implementation of these approaches might help counter recent increases in adult cyclist deaths.

The U.S. Department of Transportation’s National Highway Traffic Safety Administration (NHTSA) maintains the FARS database. FARS catalogs an annual census of fatal traffic crashes from the years 1975–2012 collected through agreements between NHTSA and agencies in each state. To be included in FARS, an incident 1) must involve a motor vehicle traveling on a roadway open to the public, and 2) must have resulted in the death of a motorist or a nonmotorist within 30 days of the crash.

This analysis uses FARS variables that were consistent during the period 1975–2012. Cyclist fatalities were identified using the “person type” descriptors “nonmotorist: pedalcyclist,” “nonoccupant bicyclist,” and “bicyclist” in the FARS “person” tables. Consistent data from the entire study period were available from 48 states (data were not available from Alaska and Hawaii) and the District of Columbia. The age and sex of the injured person as well as the state and county of the crash were collected from FARS. Annual county population, stratified by age and sex, was obtained from the National Cancer Institute’s Surveillance, Epidemiology, and End Results (SEER) program.* Age-adjusted mortality rates were calculated for each year using CDC’s published weights for the 2000 U.S. standardized population.† Data were downloaded, processed, and analyzed using generalized linear models with statistical software.

Over the 38-year study period, FARS captured 29,711 cyclist deaths. Annual cyclist fatalities declined from a high of 955 in 1975 to 717 in 2012. The annual age-adjusted mortality rate declined 44%, from a high of 0.41 per 100,000 in 1975 to 0.23 per 100,000 in 2012 (Figure 1). The proportion of cyclist deaths among all annual motor vehicle–related fatalities was highest in 1975 at 2.3%, dipped to a low of 1.4% in 2003, and increased to 2.2% by 2012 (Figure 1).

Trends in age-specific cyclist mortality rates varied in magnitude and direction (Figure 2). In bivariate linear models, mortality rates for age groups <35 years and ≥75 years decreased significantly over the study period, with the largest decrease among children aged <15 years. Historically, mortality rates for children aged <15 years were substantially higher than rates for other age groups. In 1975, the mortality rate for children aged <15 years was 1.18 per 100,000, more than four times higher than the rate (0.25 per 100,000) for persons aged ≥15 years. This pattern shifted over the 38-year study period, and by 2012, the rate among children aged <15 years (0.09 per 100,000) was one third that of all other age groups (0.27 per 100,000). During 1975–2012, the cyclist mortality rate among children aged <15 years declined 92%. The overall decrease in age-adjusted mortality rates can be attributed to declines among children aged <15 years because no linear decline was observed when children were excluded from models.

Mortality rates for adults aged 35–74 years increased significantly during the study period. The largest increase was among adults aged 35–54 years, with the mortality rate increasing nearly threefold, from 0.11 to 0.31 per 100,000.

The overall mortality rate for males was six times greater than the overall mortality rate for females. In 2012, males accounted for 87% of total bicycle deaths in the United States. This proportion increased over the 38-year study period, from 79% in 1977 to a peak of 90% in 2001.

All 48 states and the District of Columbia experienced a decrease in age-adjusted cyclist mortality rates when comparing averages during the first 5 years with those during the last 5 years of the study period (Table). Cyclist mortality rates varied more than 10-fold across jurisdictions, from a low of 0.04 per 100,000 (Vermont) to a high of 0.57 per 100,000 (Florida). Maine had the greatest decrease in cyclist mortality (78.7%) and declined from 0.47 per 100,000 to 0.10 per 100,000. Florida saw one of the smallest decreases (9.7%) in its age-adjusted cyclist mortality rate, from 0.63 to 0.57 per 100,000.

## Discussion

Overall, substantial declines have been observed in cyclist mortality, and these declines are attributable to declines in mortality among children. Changes in cyclist mortality rates vary by sex, age, and state. Many factors likely contribute to trends in bicycling fatalities, including prevalence of bicycling, road design and engineering, traffic law enforcement, driver and bicyclist behavior, helmet use, and traffic volume. Although bicycles account for a relatively small share of trips across all modes of transportation, the share of total household trips taken by bicycle has doubled over the last 35 years, and in 2009, bicycling accounted for approximately 1% of trips in the United States (4). Recent years have seen the largest increase in bicycling; for instance, during 2000–2012, the number of U.S. workers who traveled to work by bicycle increased 61% (6). This growth is not uniform because most has occurred among men aged 25–64 years, whereas cycling rates have remained steady for women and have fallen among children (4). Although many factors could influence cyclist mortality trends, the observed trends by age and sex during the study period likely reflect the changing prevalence of cycling among those groups. Thus, the decline in bicyclist mortality among children might be attributable to fewer child bicycle trips rather than a result of safer road conditions. Increased use of helmets among children might also have contributed to reduced child bicyclist mortality over the study period (7).

The findings in this report are subject to at least three limitations. First, FARS fatalities must involve a motor vehicle on a public road, so this analysis does not include cyclist fatalities in which a vehicle was not involved or which occurred off of a public road. Second, mortality rates based on population do not account for exposure to bicycling in the way that expressing deaths per unit time bicycling, distance traveled, or number of trips would. This analysis found that approximately 2% of 2009 motor vehicle–related deaths were cyclists, and data from the 2009 National Household Travel Survey suggest that travel by bicycle accounted for 0.9% of all travel time and 0.2% of all travel distance (8). Mode-specific deaths expressed per unit distance traveled or per trip would likely further highlight disparities between modes (2). Calculation and interpretation of age-specific state mortality rates were limited by the rarity of fatalities for some year-state-age group combinations. Finally, the analysis focused on long-term trends in FARS data and, therefore, did not use variables that were added in recent years. Future studies could explore recent cyclist mortality trends in greater detail by incorporating newer FARS data on crash location, road type, helmet use, distraction, or inebriation, as well as data from other sources on cycling trips and distance traveled among various age groups.

**FIGURE 1:**
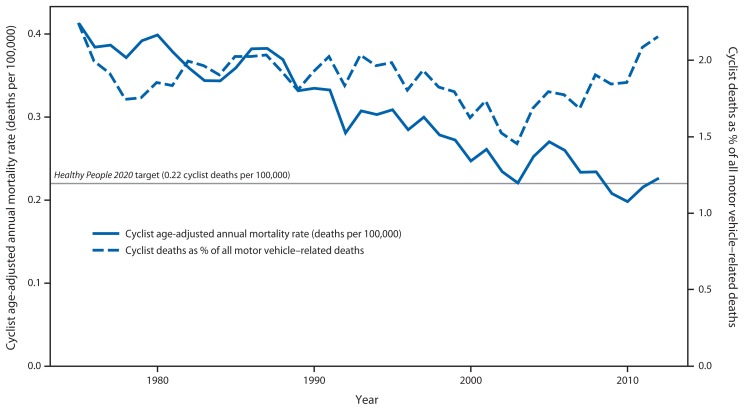
Cyclist age-adjusted annual mortality rate and cyclist proportion of all motor vehicle–related deaths — United States, 1975–2012

Public health goals of increased physical activity and population interest in alternatives to automobile transportation place additional focus on bicycle safety. Over the past decade, per capita motor vehicle travel has decreased (9), and persons have used bicycles for more utilitarian trips (e.g., commuting to work or going to the grocery store) (4,6). The reasons for these transportation shifts are multifactorial and include economic drivers, such as fuel prices and unemployment, as well as health and environmental benefits. Nonetheless, these shifts, combined with recent increases in the proportion of road deaths accounted for by cyclists (Figure 1), suggest an opportunity for expanding traditional road safety interventions in the United States (which have largely focused on vehicle passenger safety) with interventions designed to protect cyclists.

This report underscores the importance of improving bicycle safety in the United States with the aim of preventing fatalities. In addition, a common perception that cycling is unsafe might contribute to low levels of bicycling, diminishing opportunities for physical activity, particularly among women and children (10). Several countries and some U.S. cities have higher bicycle use and lower mortality rates than the United States overall. Many have implemented multifaceted, integrated approaches to bicycling that address safety while also promoting cycling (1). Such approaches often include extensive bicycle infrastructure (e.g., physically separated bike lanes), traffic calming measures (e.g., speed humps), legal interventions (e.g., lowered speed limits), travel programs (e.g., safe routes to school), and education to encourage safe bicyclist and motorist behavior (1). Other strategies that can reduce fatalities include helmet laws and improved conspicuity of cyclists via lights and bright or reflective clothing.§ Overall, cyclist mortality has decreased in recent years, but adults remain at elevated risk. Multifaceted approaches to bicycle road safety are likely needed to ensure bicycling safety for all.

**Summary**

What is already known on this topic?

On a per trip basis, bicyclists are twice as likely as vehicle occupants to die on U.S. roads. About 1% of all trips are by bicycle, and bicycling has increased recently among adults while declining among children.

What is added by this report?

During 1975–2012, overall annual rates for cyclist mortality decreased 44%, with the steepest decline among children aged <15 years. In contrast, cyclist mortality rates increased for adults aged 35–74 years, particularly men aged 35–54 years.

What are the implications for public health practice?

Multifaceted, integrated approaches to bicycling have improved safety while also promoting cycling. With cycling increasing in the United States, especially in urban areas, improving bicycle safety could prevent potential increases in cyclist mortality rates.

## Figures and Tables

**FIGURE 2:**
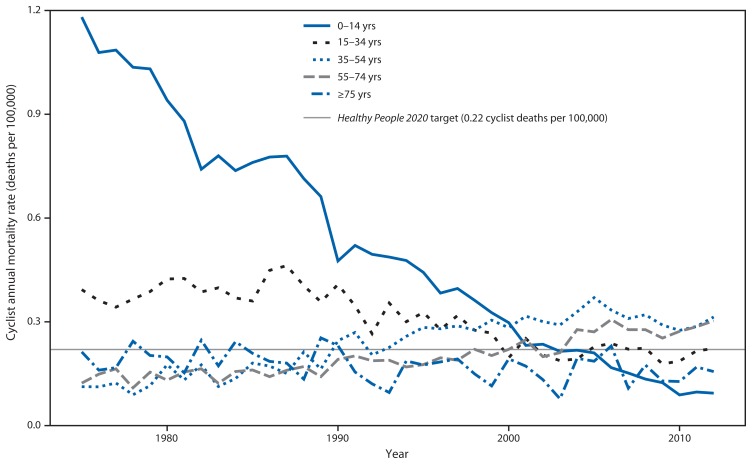
Cyclist annual mortality rates relative to the *Healthy People 2020* target, by age group — United States, 1975–2012

**TABLE:** Average annual age-adjusted cyclist mortality rates, by state* — United States, 1975–1979 and 2008–2012

State	1975–1979	2008–2012	% decrease
Alabama	0.23	0.12	48.0
Arizona	0.62	0.32	48.1
Arkansas	0.33	0.20	40.1
California	0.41	0.29	29.6
Colorado	0.31	0.20	33.9
Connecticut	0.30	0.14	51.2
Delaware	0.51	0.38	25.5
District of Columbia	0.30	0.14	53.6
Florida	0.63	0.57	9.7
Georgia	0.41	0.18	55.9
Idaho	0.39	0.20	48.9
Illinois	0.36	0.20	45.4
Indiana	0.41	0.20	52.4
Iowa	0.31	0.15	52.2
Kansas	0.34	0.17	49.3
Kentucky	0.27	0.14	48.0
Louisiana	0.50	0.33	34.4
Maine	0.47	0.10	78.7
Maryland	0.24	0.12	51.2
Massachusetts	0.29	0.13	56.9
Michigan	0.51	0.22	56.3
Minnesota	0.47	0.17	64.9
Mississippi	0.38	0.21	45.8
Missouri	0.24	0.07	71.1
Montana	0.38	0.15	60.8
Nebraska	0.29	0.08	71.9
Nevada	0.59	0.20	66.0
New Hampshire	0.32	0.11	64.2
New Jersey	0.30	0.17	45.0
New Mexico	0.33	0.27	17.9
New York	0.43	0.21	51.9
North Carolina	0.46	0.25	45.1
North Dakota	0.42	0.15	65.1
Ohio	0.32	0.14	55.4
Oklahoma	0.30	0.17	44.5
Oregon	0.48	0.26	45.9
Pennsylvania	0.30	0.11	62.9
Rhode Island	0.19	0.10	45.0
South Carolina	0.71	0.28	60.1
South Dakota	0.41	0.10	74.6
Tennessee	0.32	0.11	64.9
Texas	0.39	0.20	49.6
Utah	0.37	0.17	55.0
Vermont	0.25	0.04	82.4
Virginia	0.30	0.14	53.9
Washington	0.30	0.13	56.5
West Virginia	0.25	0.06	76.9
Wisconsin	0.52	0.16	69.4
Wyoming	0.18	0.17	6.7

* Includes 48 states and the District of Columbia (data were not available from Alaska and Hawaii).

## Footnotes

* Additional information available at http://seer.cancer.gov/popdata.

† Additional info available at http://stacks.cdc.gov/view/cdc/13357.

§ Additional information available at http://www.nhtsa.gov/staticfiles/nti/pdf/811727.pdf.
